# Physiological baseline of conscious tigers: expanded diagnostic framework integrating renal, electrolyte and cardiorenal biomarkers in *Panthera tigris*

**DOI:** 10.3389/fvets.2026.1747860

**Published:** 2026-02-26

**Authors:** Boon Allwin, Anand Rajshekhar Dadke, Kalaignan Pajaniradjou, Jayvin Hitendra Kelaiya, Digjay Vasantbhai Kabariya

**Affiliations:** Department of Wildlife Health Management, Greens Zoological Rescue and Rehabilitation Centre, Jamnagar, Gujarat, India

**Keywords:** biochemical reference, cardiorenal axis, cTnI, NT-ProBNP, *Panthera tigris*, SDMA, welfare

## Abstract

Accurate biochemical reference values for large felids have historically relied on anesthetized or stress-induced sampling, potentially confounding true physiological baselines. This study establishes the first non-anesthetic biochemical reference ranges for tigers (*Panthera tigris*), defining authentic renal, electrolyte and cardiac parameters under voluntary conscious conditions. Forty captive tigers conditioned for voluntary tail-vein blood withdrawal provided 80 samples without sedation or restraint. Analytes included renal markers (BUN, creatinine, SDMA and uric acid), electrolytes (Na^+^, K^+^ and Cl^−^), and cardiac indices (cTnI, NT-proBNP and CK-MB). Mean ± SD values were: BUN 15 ± 2 mg/dL, creatinine 1.2 ± 0.1 mg/dL, SDMA 8.0 ± 0.5 μg/dL, uric acid 4.5 ± 0.6 mg/dL, Na^+^ 151 ± 2 mEq/L, K^+^ 4.6 ± 0.4 mEq/L, Cl^−^ 111 ± 3 mEq/L, cTnI 0.02 ± 0.01 ng/mL, NT-proBNP 200 ± 25 pg./mL, and CK-MB 7.5 ± 1 U/L. Strong BUN–creatinine (r = 0.82 *p* < 0.001) and SDMA–creatinine (r = 0.80 *p* < 0.001) correlations confirmed synchronized glomerular filtration, while Na^+^–Cl^−^ coupling (r = 0.92 *p* < 0.001) validated ionic balance. Cardiac indices exhibited low variability (CV < 10%) and a moderate SDMA–NT-proBNP relationship (r = 0.46 *p* < 0.01) demonstrated a functional cardiorenal continuum. Using voluntary sampling, this study defines the first physiologically valid biochemical and cardiorenal reference baselines for tigers supporting improved welfare, greater diagnostic reliability and earlier identification of renal and cardiac pathology in managed large felids.

## 1 Introduction

The decline in tiger populations has been attributed to factors such as poaching, reduction in prey base, nutritional deficiencies and infectious diseases. Accurate biochemical baselines form the foundation of clinical diagnostics for preventive health monitoring in managed wildlife. Consequently, effective health monitoring and management along with timely disease diagnosis and appropriate treatment are critical components for the conservation and long-term sustainability of tiger populations (1). The concentrations of biochemical constituents in tissues and body fluids are normally maintained within a narrow physiological range; however, under adverse or pathological conditions these levels may become either elevated or reduced (2). Viral, bacterial and parasitic infections are commonly reported in tigers and are known to influence hematological and biochemical parameters thereby altering normal reference values (3). Despite their biological and conservation significance, age and sex related variations in the hematological parameters of Bengal tigers have not been systematically investigated (4). For large carnivores, particularly the Bengal tiger (*Panthera tigris*) most available reference data are derived from anesthetized or restrained individuals conditions that significantly alter physiological parameters (5–8). Anesthetic agents such as medetomidine and ketamine exert well-documented effects on cardiac output, glomerular filtration rate (GFR) and electrolyte regulation introducing substantial artefactual variability that compromises diagnostic accuracy and confounds welfare assessment (5–8). These drug-induced physiological perturbations obscure true baseline function, limiting the interpretability of renal and cardiovascular biomarkers.

In contrast, sampling performed under conscious, low-stress conditions provides a physiologically representative assessment of renal, cardiac and metabolic status free from anesthesia-related bias. This distinction is particularly critical in tigers, a species predisposed to renal dysfunction associated with dehydration, high-protein diets and age-related nephrosclerosis (9–12). Despite the clinical relevance of cardiovascular parameters in this context, cardiac indices in tigers remain poorly characterized, largely due to reliance on anesthetised measurements. Unanesthetised sampling therefore represents an essential methodological advance to accurately characterize baseline physiology, improve diagnostic precision and enhance welfare-relevant research outcomes in this species.

This study describes a welfare-centric diagnostic framework by conditioning tigers for voluntary tail-vein blood collection eliminating the need for sedation. It integrates conventional biochemical indices with novel biomarkers Symmetric Dimethylarginine (SDMA), N-terminal pro–B-type natriuretic peptide (NT-proBNP) and cardiac troponin I (cTnI) to elucidate interconnections between renal and cardiac physiology. The work aims to establish the first authentic physiological baselines for tigers under true resting conditions and to demonstrate the diagnostic potential of unanesthetised sampling for early detection of cardiorenal compromise in large felids.

## 2 Materials and methods

Animals and Welfare Conditioning: Forty adult Bengal tigers (*Panthera tigris*) comprising 20 males and 20 females (aged 4–16 years) were included in the study. All individuals were maintained at the Greens Zoological Rescue and Rehabilitation Centre (GZRRC), Jamnagar, Gujarat, India. The animals were housed in enclosures consisting of indoor and semi-outdoor sections with a squeeze cage positioned between the two areas. The semi-outdoor enclosure was connected to an adjoining large paddock area. As part of standard operating procedures for enclosure management animals were routinely shifted from the indoor enclosure to the semi-outdoor area daily both before and after feeding to facilitate enclosure cleaning and husbandry activities. The squeeze cage measured 1.63 m × 1.07 m × 1.23 m (length × width × height). This routine movement also served to gradually acclimatize the animals to the squeeze cage thereby promoting voluntary compliance. During blood collection, animals were positioned comfortably within the squeeze cage. Positive reinforcement was used, allowing controlled access to the tail through the gap between the squeeze cage panels. Once the tail was secured a tourniquet was applied proximal to the intended venipuncture site and the area was aseptically prepared using clinical spirit. Blood samples were collected from the lateral coccygeal vein. The overall procedure remained well tolerated by the animals and repeatable ensuring both animal welfare and handler safety.

During blood collection, the animal demonstrated a calm and composed demeanor while restrained in the squeeze cage. Positive reinforcement techniques including gentle handling and familiar cues were employed to promote voluntary cooperation and reduced anxiety. The animal showed minimal behavioral signs of stress such as vocalization, struggling or defensive posturing and remained relaxed throughout the procedure. This calm behavior facilitated smooth venipuncture and ensured the safety of both the animal and the handlers while also reflecting effective acclimatization and humane handling practices that support animal welfare. This conditioning protocol eliminated the need for anaesthesia.

### 2.1 Sample collection and processing

Approximately 3 mL of blood was collected from the lateral tail vein into plain vacutainer tubes. Samples were allowed to clot centrifuged at 3,000 rpm for 10 min and analysed within 2 h of collection to preserve biochemical integrity.

### 2.2 Biochemical analyses

Serum samples were analysed for multiple biochemical and clinical parameters using standardized veterinary diagnostic platforms. Blood urea nitrogen (BUN), creatinine and uric acid were estimated by colorimetric dry chemistry slide methods, while electrolyte concentrations - sodium (Na^+^), potassium (K^+^) and chloride (Cl^−^) were determined using a potentiometric dry chemistry technique all performed on the FUJIFILM Dry-Chem NX700 analyser. SDMA concentrations were measured using a competitive immunoassay with a mouse monoclonal anti-SDMA antibody and cardiac biomarkers including NT-proBNP and cTnI were quantified using a fluorescent immunoassay these analyses were carried out on the BIONOTE Vcheck V200 system. Creatine kinase-MB (CK-MB) activity was also assessed by a colorimetric method.

### 2.3 Statistical analyses

The data obtained from both male and female tigers were subjected to statistical analysis using two-way analysis of variance (ANOVA). Statistical analyses were performed using the WASP (ICAR) statistical software to evaluate the correlation coefficients and other relevant parameters. This analysis was performed to compare the mean values of each biochemical and haematological parameter between sexes. Pearson’s correlation coefficients (r) were calculated for all paired biochemical parameters. Statistical significance of correlations was assessed using two-tailed tests, and *p*-values < 0.05 were considered statistically significant.

## 3 Results

### 3.1 Renal axis and SDMA sensitivity

In the present study, mean BUN (15 ± 2 mg/dL) and creatinine (1.2 ± 0.1 mg/dL) concentrations produced a physiologically appropriate BUN:creatinine ratio of approximately 15:1, consistent with normal nitrogen metabolism and adequate hydration status in obligate carnivores. Biochemical profiling of conscious tigers further revealed uric acid (4.5 ± 0.6 mg/dL) and electrolyte concentrations within expected physiological ranges, including sodium (151 ± 2 mEq/L), potassium (4.6 ± 0.4 mEq/L) and chloride (111 ± 3 mEq/L). All parameters remained within the expected reference ranges for large felids with minimal inter-individual variation and corroborated with published reference ranges (13, 14). Mean SDMA concentrations (8.0 ± 0.5 μg/dL) demonstrated stability across age and sex indicating potential for establishing species-specific reference intervals.

### 3.2 Cardiac biomarkers and clinical scope

Mean concentration of NT-proBNP (200 ± 25 pg/mL), cTnI (0.02 ± 0.01 ng/mL), and CK-MB (7.5 ± 1.0 U/L) in clinically normal tigers exhibited minimal inter-individual variability suggesting stable baseline cardiac biomarker profiles under the study conditions. These values align with reported ranges in other large felids, suggesting conserved cardiac biochemical baselines across the group (15).

## 4 Discussion

### 4.1 Renal axis and SDMA sensitivity

The BUN–creatinine relationship reflects intact renal perfusion and protein metabolism with negligible extrarenal influence (16). The mean uric acid concentration aligns with typical mammalian purine metabolism in carnivores where uric acid is a minor excretory product compared to urea. In tigers, uric acid remains a sensitive but secondary indicator of renal tubular excretion efficiency (17). Values obtained in conscious tigers eliminate pharmacologic confounders and therefore represent true physiological baselines critical for identifying early CKD trends over time.

The narrow standard deviations observed across the cohort indicate physiological stability during sampling in a fully conscious state suggesting that restraint-related stress did not significantly alter electrolyte or uric acid homeostasis. The observed correlation between SDMA and creatinine (*r* = 0.80) supports their shared dependence on glomerular filtration kinetics; however, the tighter variance of SDMA values underscores its superior analytical precision and sensitivity to early nephron dysfunction (18).

The renal axis is central to metabolic and physiological homeostasis in *Panthera tigris*, integrating nitrogen turnover, osmotic regulation, and electrolyte balance (19). Within this system, the BUN–creatinine–SDMA triad functions as a sensitive, multifactorial indicator of renal performance, reflecting coordinated glomerular and tubular processes essential to metabolic clearance (20). The integration of SDMA substantially enhances renal diagnostic sensitivity. SDMA, a methylated arginine derivative released during protein turnover is almost exclusively eliminated by glomerular filtration and remains independent of muscle mass a limitation inherent to creatinine-based assessments (21, 22). This property is particularly relevant in tigers, where sexual dimorphism and muscle mass variability can obscure early renal decline when relying on creatinine alone (23).

SDMA therefore serves as an effective early biomarker for preclinical renal compromise, preceding detectable creatinine elevations (3, 20). Collectively, these findings emphasize the diagnostic value of the renal axis as an integrated indicator of physiological stability and renal reserve. The inclusion of SDMA strengthens early detection frameworks for chronic kidney disease (CKD) and subclinical nephropathy in *Panthera tigris*, facilitating earlier intervention improved hydration management and long-term conservation health monitoring. A diagnostic framework for interpreting SDMA and creatinine concentrations in Bengal tigers is presented in Table 1, enabling assessment of renal function and evidence-based clinical decision-making. Normal renal filtration is indicated by SDMA values <9–10 μg/dL with creatinine within reference limits. Mild SDMA elevation in the presence of normal creatinine reflects early nephron stress or subclinical renal dysfunction, warranting hydration optimization and dietary management. True renal insufficiency is characterized by concurrent elevations in SDMA (>10 μg/dL) and creatinine (>1.4 mg/dL), indicating compromised glomerular filtration and necessitating further diagnostic evaluation, including ultrasonography and targeted therapeutic intervention. Persistently elevated SDMA (>11 μg/dL) with stable creatinine is consistent with compensated chronic nephron loss, for which serial monitoring and dietary protein restriction are recommended.

**Table 1:** Diagnostic interpretation of SDMA and creatinine in Bengal tigers.

Diagnostic scenario	Biomarker pattern	Interpretation	Recommended action
Normal filtration	SDMA < 9 μg/dL, Creatinine 1.0–1.3 mg/dL	Physiological baseline	Routine monitoring
Early renal stress	SDMA 9–10 μg/dL, Creatinine normal	Subclinical nephron strain	Hydration & diet correction
True renal insufficiency	SDMA >10 μg/dL, Creatinine >1.4 mg/dL	Compromised filtration	Ultrasound & therapy Urine Biochemistry and Specific Gravity
Chronic nephron loss	SDMA >11 μg/dL, Creatinine stable	Compensated CKD	Repeat testing; low-protein diet

### 4.2 Cardiac biomarkers and clinical scope

The narrow variance across individuals indicates hemodynamic stability and the absence of acute or chronic myocardial strain. Correlational analysis among the three biomarkers (r = 0.42–0.51) delineates a strain–injury continuum where mild elevations in NT-proBNP may precede subtle cTnI increases signaling early compensatory adaptation rather than irreversible damage. The correlation matrix defines functional interdependence among renal, cardiac, and electrolyte parameters, outlining the biochemical architecture of homeostasis in Bengal tigers. These relationships identify how metabolic, renal and cardiovascular systems co-regulate under conscious physiology. Cardiac biomarkers provide quantitative insight into myocardial health and hemodynamic balance, offering a functional assessment of cardiac performance. In *Panthera tigris*, where clinical access is often limited and stress-induced cardiophysiological changes can confound interpretation these markers provide a highly informative index of cardiac performance. The NT-proBNP–cTnI–CK-MB triad provides a layered interpretive framework encompassing myocardial load, injury and recovery. NT-proBNP, a cleavage product of pro–B-type natriuretic peptide reflects ventricular wall stress and volume overload serving as a quantitative proxy for cardiac strain and diastolic dysfunction. In contrast, cTnI represents a high-specificity biomarker of myocardial cell membrane damage responding sensitively to ischemic or stress-induced cardiomyocyte injury (23). CK-MB though less specific provides complementary data on cardiomyocyte enzyme leakage and post-injury recovery kinetics, bridging the temporal gap between acute release and systemic clearance (24).

A reference-based interpretative framework for cardiac biomarkers in Bengal tigers is presented in Table 2, correlating biomarker concentrations with underlying cardiac physiology and associated clinical recommendations. NT-proBNP values of 150–250 pg./mL reflect normal ventricular preload and support routine cardiac surveillance, whereas concentrations >300 pg./mL indicate ventricular overload, warranting heightened anesthetic caution and fluid management optimization. cTnI concentrations <0.03 ng/mL signify myocardial stability, for which continued electrocardiographic monitoring is adequate. In contrast, cTnI values >0.06 ng/mL suggest myocardial injury and necessitate further diagnostic evaluation, particularly echocardiography. Collectively, these findings substantiate the clinical value of integrated cardiac biomarker panels in large felids, reinforcing their role in proactive cardiovascular monitoring, anesthetic risk stratification, and health optimization within conservation medicine programs.

**Table 2:** Reference interpretation of cardiac biomarkers in Bengal tigers.

Biomarker	Physiological range	Interpretation	Clinical guidance
NT-proBNP	150–250 pg./mL	Normal preload	Routine screening
NT-proBNP	>300 pg./mL	Ventricular overload	Pre-anesthetic caution; fluid optimization
cTnI	<0.03 ng/mL	No myocardial stress	Stable myocardium ECG Monitoring
cTnI	>0.06 ng/mL	Myocardial injury	Echocardiography required
CK-MB	6–9 U/L	Physiologic muscle tone	Normal activity
CK-MB	>10 U/L	Muscle exertion/trauma	Re-evaluate post-handling

### 4.3 Diagnostic flow and clinical integration

The diagnostic flow integrates renal, cardiac, and electrolyte parameters into a tiered interpretation model for comprehensive tiger health evaluation. Each axis reflects a physiological checkpoint—from filtration to preload to ionic compensation.

### 4.4 Implications for big cat renal, cardiac, and metabolic health

This study establishes non-anesthetic baseline values for electrolyte (Na^+^, K^+^, Cl^−^) and uric acid parameters in conscious tigers, providing direct insights into hydration status, acid–base regulation and redox metabolism previously inferred from anesthetized or domestic carnivores. The strong Na^+^–Cl^−^ covariance (r = 0.92) confirms preserved tubular integrity, while the moderate association between uric acid and BUN (r = 0.58) reflects its integrated role in nitrogen metabolism and antioxidant defense. Together, these findings define a physiological metabolic framework linking renal filtration, osmotic control, and oxidative status under anesthesia-free conditions.

Integrated and physiologically coherent network of relationships between renal, metabolic, electrolyte, and cardiac biomarkers, reflecting real-world organ crosstalk rather than isolated laboratory values are recorded in Table 3. The strong positive correlations among BUN, creatinine, and SDMA (r = 0.75–0.82; *p* < 0.001) confirmed tightly coupled nitrogen handling and glomerular filtration, reinforcing their reliability in distinguishing true azotemia from transient biochemical fluctuations and in detecting early renal impairment independent of muscle mass. The moderate association between uric acid and BUN (r = 0.58; *p* < 0.001) suggested shared metabolic and oxidative pathways, which were accentuated during increased protein load or catabolic states. Electrolyte relationships further supported renal tubular homeostasis, with a very strong Na^+^–Cl^−^ correlation (r = 0.92; *p* < 0.001) indicating preserved tubular integrity, while the inverse Na^+^–K^+^ correlation (r = −0.36; *p* < 0.05) reflected hormonally regulated ionic balance mediated by aldosterone. Importantly, the moderate correlations linking SDMA with NT-proBNP, cTnI with NT-proBNP, and CK-MB with cTnI (r = 0.42–0.51) revealed a biologically plausible cardiorenal–cardiac continuum, in which early renal stress paralleled myocardial load and subtle injury, allowing differentiation between physiological adaptation and emerging pathology. All reported correlations were statistically significant (*p* < 0.05) reinforcing that the observed renal, electrolyte and cardiorenal associations represent true physiological relationships rather than random variation.

**Table 3:** Correlation matrix of biochemical and cardiac biomarkers.

Biomarker pair	r	Relationship	Interpretation	Clinical relevance
BUN–Creatinine	r = 0.82 *p* < 0.001	Strong +	Stable nitrogen metabolism and filtration	Parallel rise = true azotemia
SDMA–Creatinine	r = 0.80 *p* < 0.001	Strong +	Filtration synchrony; muscle independent	Detects early renal loss
BUN–SDMA	r = 0.75 *p* < 0.001	Strong +	Nitrogen–filtration concordance	Identifies metabolic dehydration
Uric Acid–BUN	r = 0.58 *p* < 0.001	Moderate +	Oxidative–metabolic coupling	High both = protein excess
Na^+^–Cl^−^	r = 0.92 *p* < 0.001	Very strong +	Tubular integrity	Deviation <0.8 = acidosis
Na^+^–K^+^	r = − 0.36 *p* < 0.05	Moderate – (Inverse Correlation)	Consistent with Aldosterone mediated ionic regulation	Maintains osmotic balance
SDMA–NT-proBNP	r = 0.46 *p* < 0.01	Moderate +	Cardiorenal link	Early systemic load detection
cTnI–NT-proBNP	r = 0.42 *p* < 0.05	Moderate +	Strain–injury link	Myocardial stress continuum
CK-MB–cTnI	r = 0.51 *p* < 0.01	Moderate +	Muscle–cardiac synchrony	Differentiates exertion vs. pathology

Table 4 presents an integrated, stepwise diagnostic flow model for comprehensive clinical assessment of tigers, linking key physiological axes to targeted clinical decision-making. The framework begins with renal axis evaluation (SDMA, creatinine, BUN) to assess filtration efficiency and nephron integrity, guiding hydration correction and renal-specific interventions. The second step addresses the cardiac axis by integrating NT-proBNP, cTnI, and CK-MB to evaluate ventricular load and myocardial injury, informing anesthetic risk stratification and the need for advanced cardiac imaging. Electrolyte assessment (sodium, potassium, chloride) follows to evaluate osmotic and acid–base balance, supporting fluid therapy decisions and endocrine monitoring. The final step emphasizes longitudinal biomarker trending- particularly SDMA and NT-proBNP to enable early detection of disease progression and support structured surveillance for proactive health management.

**Table 4:** Integrated diagnostic flow model for tiger clinical assessment.

Step	Axis/parameters	Interpretive focus	Clinical decision
Step 1	Renal (SDMA–Creatinine–BUN)	Filtration efficiency; nephron integrity	Hydration correction; renal therapy
Step 2	Cardiac (NT-proBNP–cTnI–CK-MB)	Ventricular load and myocardial injury	Adjust anesthetic plan; cardiac imaging
Step 3	Electrolytes (Na^+^–K^+^–Cl^−^)	Osmotic and acid–base balance	Fluid replacement; monitor adrenal function
Step 4	Temporal monitoring	Trend-based disease evolution	Quarterly SDMA & NT-proBNP tracking

Integrating SDMA with conventional renal indices expands the diagnostic precision of kidney health monitoring, while NT-proBNP and cTnI provide parallel insights into myocardial strain and cardiomyocyte integrity. The moderate SDMA–NT-proBNP correlation (r = 0.46) establishes a functional cardiorenal axis - a biologically significant relationship never quantified in big cats. This relationship highlights the necessity of interpreting renal and cardiac systems as a unified physiological continuum.

Clinically, this study provides a tri-axial diagnostic model encompassing:

1. Renal filtration efficiency (SDMA, creatinine and BUN).
2. Cardiac functional load and injury (NT-proBNP, cTnI and CK-MB).
3. Electrolyte and metabolic homeostasis (Na^+^, K^+^, Cl^−^ and uric acid).

This model enables early detection of renal or cardiac compromise facilitates pre-anesthetic risk profiling and enhances welfare monitoring for geriatric, breeding and translocated tigers. Beyond veterinary application the findings bridge One Health physiology and conservation management.

In summary, this is the first report to define authentic anesthesia-free biochemical baselines for renal, cardiac, electrolyte and uric acid physiology in Bengal tigers. The study introduces both a novel methodology and a multidimensional diagnostic continuum that integrates filtration, myocardial performance and metabolic balance. Together, these contributions mark a significant advancement in big cat healthcare transforming it from periodic, anesthesia-dependent assessments to continuous, evidence-driven and welfare-positive health surveillance.

## Data Availability

The raw data supporting the conclusions of this article will be made available by the authors, without undue reservation.
